# Short Duration Repetitive Transcranial Electrical Stimulation During Sleep Enhances Declarative Memory of Facts

**DOI:** 10.3389/fnhum.2019.00123

**Published:** 2019-04-12

**Authors:** Nicola Cellini, Renee E. Shimizu, Patrick M. Connolly, Diana M. Armstrong, Lexus T. Hernandez, Anthony G. Polakiewicz, Rolando Estrada, Mario Aguilar-Simon, Michael P. Weisend, Sara C. Mednick, Stephen B. Simons

**Affiliations:** ^1^Department of General Psychology, University of Padova, Padova, Italy; ^2^Department of Cognitive Science, University of California, Irvine, Irvine, CA, United States; ^3^Teledyne Scientific & Imaging, Durham, NC, United States; ^4^Rio Grande Neurosciences, Dayton, OH, United States

**Keywords:** declarative, memory consolidation, sleep, slow oscillation, stimulation, tDCS

## Abstract

Transcranial electrical stimulation (tES) during sleep has been shown to successfully modulate memory consolidation. Here, we tested the effect of short duration repetitive tES (SDR-tES) during a daytime nap on the consolidation of declarative memory of facts in healthy individuals. We use a previously described approach to deliver the stimulation at regular intervals during non-rapid eye movement (NREM) sleep, specifically stage NREM2 and NREM3. Similar to previous studies using tES, we find enhanced memory performance compared to sham both after sleep and 48 h later. We also observed an increase in the proportion of time spent in NREM3 sleep and SDR-tES boosted the overall rate of slow oscillations (SOs) during NREM2/NREM3 sleep. Retrospective investigation of brain activity immediately preceding stimulation suggests that increases in the SO rate are more likely when stimulation is delivered during quiescent and asynchronous periods of activity in contrast to other closed-loop approaches which target phasic stimulation during ongoing SOs.

## Introduction

During sleep, the brain reprocesses and reorganizes prior learning in a memory consolidation process by which labile information becomes stronger, more efficient, and more resistant to interference (for a review see Rasch and Born, 2013). Non-rapid eye movement (NREM) sleep, which can be divided into three stages, NREM1, NREM2, and NREM3, has been recognized to benefit declarative (explicit) memory consolidation; with specific brain oscillations during NREM sleep playing an important role (Diekelmann and Born, 2010; Rasch and Born, 2013; Staresina et al., 2015). Critical among these are slow oscillations (SOs) which dominate NREM3 (slow wave) sleep and a significant body of research has investigated their role in learning and memory (Rasch and Born, 2013).

Enhancing SOs during sleep is thought to improve subsequent memory performance by enhancing the strength or coupling between memory-related, nested brain oscillations, including sigma frequency band (12–15 Hz; Cellini and Capuozzo, 2018; Wilckens et al., 2018). Non-invasive transcranial electrical stimulation (tES) has been applied as an intervention to enhance SO activity. For example, Marshall et al. (2006) demonstrated that slow oscillatory transcranial direct current stimulation (tDCS) increased SO activity as well as spindle activity, and in turn enhanced performance on a paired-association task. A recent meta-analysis of studies involving tDCS, transcranial alternating current stimulation (tACS), and slow oscillatory tDCS during sleep indicates that tES is effective in modulating declarative memory consolidation (Barham et al., 2016). Previous studies using tES during sleep typically stimulate for several minutes once an individual has been confirmed to be in NREM2 or NREM3 sleep (e.g., Marshall et al., 2006 applied 5 min of continuous 0.75 Hz slow oscillatory tDCS after 4 min of NREM2 sleep).

Recent studies also suggest that the timing of stimulation may be important for optimal enhancement of memory-related brain oscillations. For example, closed-loop sensory stimulation that delivers brief auditory stimulation during the positive peak of the SO results in a beneficial memory effect (Ngo et al., 2013; Santostasi et al., 2015; Ong et al., 2016; Leminen et al., 2017; Papalambros et al., 2017). These studies have shown an increase in SO power and phase-locked spindle activity during the up-state of the SO, and an improvement in subsequent declarative memory performance (for a review see Cellini and Mednick, 2019).

Here, we sought to determine whether short durations of repetitive tDCS delivered throughout NREM2/NREM3 could similarly be used to enhance declarative memory. Such a paradigm offers a couple of potential advantages over the more standard minutes-long paradigm used by Marshall et al. (2006). First, it offers an opportunity to more regularly and consistently investigate the brain’s acute response to the stimulation. Second, it can potentially reduce the overall dose of stimulation delivered to the individual.

Here, we tested this variation of sleep-based short duration repetitive-tES (SDR-tES) after learning in a novel, ecologically valid, a task that required participants to learn a series of facts presented in a paradigm similar to classroom learning or studying flash cards. We find that this intermittent, short-duration tES can replicate the critical effects of tES on memory and sleep. Specifically, when delivered during a nap taken after a declarative memory task it leads to both: an increase in the proportion of time spent in NREM3 sleep, and a concurrent improvement in longer-term (48-h post-test) recall of facts. Physiologically, we find that this type of stimulation boosts the rate of SOs during non-stimulation intervals. No significant changes were noted in phase-locked sigma activity between stimulation and sham. Retrospective analysis of electroencephalography (EEG)-measured brain activity immediately preceding stimulation suggests a negative correlation between the strength and coherence of local brain activity and the resulting number of SO following stimulation.

## Materials and Methods

### Participants

The study was approved by the New England Independent Review Board. An independent review board was used because the study was led by Teledyne and not the partnering academic institution. Potential participants were screened through an online survey (SONA systems) requiring them to answer questions about their sleep history and any potentially disqualifying medical or lifestyle conditions before being allowed to participate in any study session. In particular, participants were screened to ensure that proper sleep habits were engaged in leading up to the day of the study to prevent enrollment or continuation of participants that were sleep restricted or deprived. After enrollment or disqualification responses were discarded. Participants were excluded if they indicated that they were not between the ages of 18–50 years old; had any medical or neurological diagnosis including and especially sleep disorders or indications of extreme fatigue or motor coordination; had any psychiatric diagnosis requiring medication or hospitalization or that caused disability; a first-degree relative with a psychiatric disease requiring medication or hospitalization; had undergone any recent hospitalizations; had any contraindications to tES or magnetic resonance imaging (MRI); used narcotics or psychotropic medications or illicit drugs; consumed more than 10 alcoholic beverages per week; and/or had unusual work hours (e.g., night shift). Potential participants were also asked to complete the Center for Epidemiologic Studies Depression Scale (CES-D) questionnaire to screen for possible depression (Radloff, 1977). If the potential participant’s score was 16 or greater, the person was excluded.

If the person passed the initial screening, they were allowed to sign up for study sessions. They were instructed to avoid caffeine, alcohol, or naps within 24 h of any experimental session and to engage in proper sleep habits. During their first visit, all participants gave written informed consent in accordance with the Declaration of Helsinki. A total of 57 individuals (28 women) were recruited from the surrounding communities in Durham, NC; Dayton, OH; and Riverside, CA, out of which 17 individuals (six women, M_age_ = 32.24 years, SD_age_ = 8.06 years) successfully completed the necessary experimental sessions. The remaining participants were excluded from subsequent analyses due to one or a combination of the following: (1) they did not complete both the sham and SDR-tES conditions (i.e., they were unable to nap, or unable to attend all scheduled sessions); (2) fewer than 20 stimulation deliveries during an intervention session; (3) fewer than 45 min of the nap was spent in NREM2 or NREM3; and/or (4) the presence of excluding behavior or characteristics was discovered during the study (e.g., worked at night and typically slept during the day). Table 1 provides the demographic data of the included participants. The high exclusion rate in our study is a product of our within-subjects design which requires participants to come in for multiple days across 2 weeks and successfully nap in the lab on two separate occasions.

**Table 1:** Participant demographics.

	Participants (*N* = 17)
Mean age (SD)	32.24 years (8.06)
Number of women	6
Ethnicity	
White/Caucasian	14
Black/African American	3
Education	
Less than high school	-
High school	-
Some college	5
Associate degree	6
Bachelor degree	4
Graduate degree	2

*SD, standard deviation*.

Our final participant distribution concluded with an uneven distribution in sex with more males (11) completing the study than females (6). However, no significant differences were identified in measures of memory recall at any measurement time (no main effect of sex, *F*_(1,16)_, *p* = 0.165, and no interactions of test time or intervention condition, *F*_(1,16)_, all *p* > 0.36). There were also no identified sex differences in sleep architecture (i.e., NREM3 time, *t*_16_, all *p*’s > 0.24).

### Procedure

**Figure 1:**
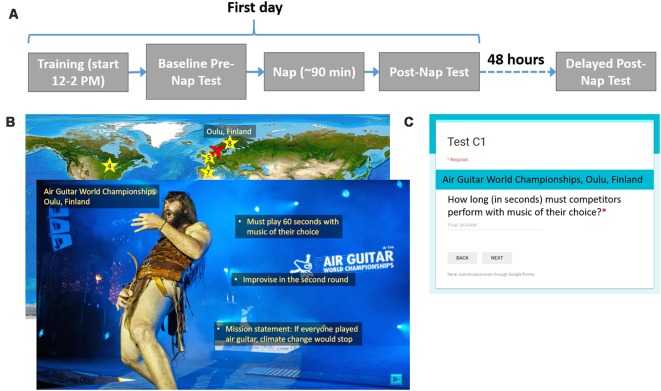
Procedure and example training and testing materials. **(A)** For each experimental condition, participants were asked to complete two visits as shown. On the first day, participants learned about 20 locations, took an immediate test, and then attempted a 90-min nap. After a 30-min sleep inertia break, participants took the first post-nap test. About 48 h later, participants returned for the final post-nap test. **(B)** Participants saw a map of the world(“Large World Map bright” image created by user MTBlack and made available under an Attribution-Share Alike 4.0 International license at https://commons.wikimedia.org/wiki/File%3ALarge_World_Map_bright.jpg)with destinations marked. The overlaid slide shows a photo (Photo Credit: Juuso Haarala and the Air Guitar World Championships. Permission for use granted by the Air Guitar World Championships) of a given location with all three facts superimposed on it. **(C)** This image shows the general format for each test question.

In a within-subjects design, participants received both SDR-tES and sham conditions. The first days of each experimental condition were separated by at least 1 week, and the order or condition was counterbalanced across subjects. Figure 1 shows the procedure for one experimental session. On the first day between 12:00 and 2:00 PM, participants were instrumented with EEG, electrocardiogram (ECG), and stimulation electrodes. The sets of material, the versions of the set, and the order of interventions were counterbalanced across participants. Participants completed the training task on a standard desktop environment and then took the baseline recall test. Before the nap, electrooculography (EOG) electrodes were placed at the corners of the eyes. Additionally, all participants were instrumented with stimulation electrodes and underwent the stimulation acclimation procedure regardless of treatment condition. The participants were allowed 90 min to sleep from the time they initially fell asleep. In the sham condition, the stimulation device was powered on but the software was turned off so that stimulation would not be triggered. After the nap, a 30-min break was imposed in order for the participant to overcome any sleep inertia, during which time participants completed a demographics questionnaire, the Big Five Inventory (John et al., 1991), and the State-Trait Anxiety Inventory Y-2 (Spielberger, 1983). After the sleep inertia break, participants took the first post-nap test. Before leaving, participants were given an actigraphy device to track their overnight sleep, or if one was not available, asked to keep track of hours slept so they could report it when they returned for the final test. About 48 h later, participants returned for the delayed post-nap test. These procedures were repeated for the both experimental sessions.

### Fact-Learning Task

The learning task consisted of remembering facts from different, relatively obscure locations around the world. Three sets of material, consisting of 20 locations each, were developed so that a within-subjects study could be conducted. For each set, three versions were created in which the order of the 20 locations was randomized. Microsoft Powerpoint was used to present the information.

Before training began, participants were told that they would learn three facts about each location and that they should read and listen carefully since they would be tested on the facts later. To give participants the opportunity to take a break, the participants learned facts about the first 10 locations, took a self-timed break, and then learned about the last 10 locations. The entire presentation was self-paced except for a minimum time constraint imposed for each slide to ensure that the participant could not effectively skip slides by clicking too quickly. Each location was associated with a unique, congruent sound cue. For example, the Air Guitar World Championships in Oulu, Finland, was paired with the sound of an electric guitar. The sounds were developed as a part of the protocol in order to enable the use of the targeted memory activation approach which was not used in this study. We include the description of them here for completeness.

For each location, a world map appeared with an airplane icon located at the previous location they had just learned about (for the first location the airplane started at Washington DC). The airplane was animated to move from the previous location to the present destination on a world map in order to direct participants’ attention to its location. Once the airplane reached its destination, a star appeared as a location marker, along with the name of the city or region and the country. Next, participants saw a photo of the location with the name of the specific place within the city or region if applicable, the city or region, and the country, lasting 2.5 s before they could choose to move on. Next, they listened to a short narration about the location that included the three relevant facts. All narration was recorded by the same female speaker. Finally, participants read the three facts, one at a time, which were overlaid onto the photo. Each fact appeared for 5 s before the participant was allowed to move on to the next fact. Facts appearing earlier remained on the screen as newer ones appeared. The associated sound cue with each location was played a total of five times: after the airplane moved to its destination on the map, when the photo and location name first appeared, and during the appearance of each of three facts. After each participant’s self-timed break, this process repeated for the other 10 locations in the set of 20. A small set of pixels at the top right corner of each slide were coded at varying grayscale values. A photosensor (Brain Products GmbH, Munich, Germany) was attached to the screen with an adhesive to measure the intensity of the pixels, allowing for timing information of the material to be recorded. Although participants were allowed to self-pace their time through the facts, we found that the allotted 5 s was more than enough time for most participants to read each fact and there were no significant differences in mean time spent per slide across locations between conditions (7.1 s ± 1.6 vs. 6.86 s ± 1.52 for sham and stimulation conditions, respectively).

There were three memory tests in total: a baseline test given immediately after training, a post-nap test administered 30 min after the participant woke up to reduce the effects of sleep inertia, and a delayed test given about 48 h later. Each test consisted of 20 questions, each question corresponding to a fact from one of the 20 locations. None of the questions were repeated across testing intervals. Google Forms was used to present each question with a field for the participant to type in the answer. The name of the location always appeared above the question. Questions were presented one at a time. Participants could not continue to the next question until an answer was entered and could not return to previous questions once they had moved on. They were instructed to be as specific as possible in their answers, and discouraged from putting answers such as, “I don’t remember” or “I don’t know.” Tests were scored by one of the researchers. Figure 1 shows the procedure for one experimental session, examples of training material for one location, and the format of each test question.

Memory performance was computed for each participant as a percentage of correctly recalled facts at each testing session relative to the percentage of correctly recalled fact during the baseline.

### Electrophysiological Recording

EEG data were collected on a Brain Products 32-channel actiCAP electrode system and BrainAmp DC amplifier (Brain Products, GmbH, Munich, Germany) using the standard 10–20 electrode layout. ECG and EOG electrodes were used for offline artifact rejection and assessment of REM sleep. For EOG, one electrode was placed 1 cm above the corner of the right eye and the second electrode was placed 1 cm below the corner of the left eye following recommended criteria for sleep recording (Berry et al., 2012). The left shoulder blade was used as a common reference. EOG was collected to facilitate offline sleep scoring used in the analysis of NREM sleep biomarkers and for reference against automated sleep scoring in our system. Active reference electrodes were attached to the left and right mastoid sites with an adhesive ring. All electrophysiological data were collected at a sample rate of 500 Hz and recorded using the Open Vibe software^1^ for offline analysis. The frequency boundaries during recording were 0–1,000 Hz.

### Short-Duration Repetitive Transcranial Electrical Stimulation

The electrode locations and the stimulation waveform (except duration and delivery approach) were identical to those in Marshall et al. (2006). Two Ag/AgCl stimulation electrodes in custom-made plastic holders with a 16-mm diameter each were filled with conductive gel (Signagel, Parker Laboratories, Inc.) and placed anterior and posterior to bilateral frontal positions F3 and F4 and at the mastoids, for a total of eight electrodes on the head. Differences in the electrodes used required this configuration with multiple electrodes to approximate the delivered current density reported in Marshall et al. (2006; i.e., 0.517 mA cm^–2^). Four seconds of 0.75-Hz oscillating current was delivered using a battery-driven, constant current NeuroMod16 device (Rio Grande Neurosciences, Inc., Dayton, OH, USA). Before every nap, an acclimation procedure was performed such that the waveform was delivered at least twice at half the maximum current intensity (1.0 mA) and then the desired maximum current intensity (2.0 mA) to ensure participant comfort. Participants provided pain ratings on a scale from 0 (no pain at all) to 10 (worst pain imaginable). Participants that rated the stimulation above a four rating (mildly uncomfortable) were to be excused from any further participation; however, no participants gave ratings higher than 4. During the sham session, electrodes were applied as in the real stimulation sessions, the stimulation device was turned on, and the acclimation procedure was completed, but no stimulation was delivered during the nap.

**Figure 2:**
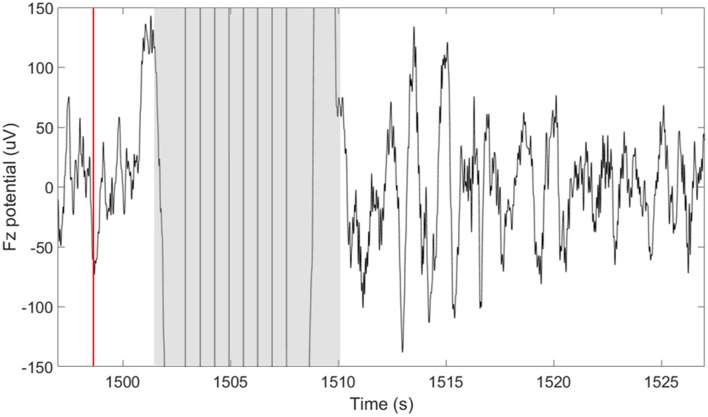
Short-duration delivery of slow oscillatory transcranial direct current stimulation (tDCS) increases the subsequent slow oscillations (SOs) rate. The plot shows the potential measured at the frontal midline electrode (Fz) 30 s around the time of a single stimulation cycle. The time of the down-to-up phase detection is shown with the red line at ~1,498 s. The time of stimulation including the excluded recovery period are highlighted in gray and span approximately 8 s (1,502–1,510 s). A large number of SOs are observed in the period following the stimulation.

TES delivery was intermittently triggered based on real-time detection of SOs using our closed-loop system described in detail in Shimizu et al. (2018). In summary, this system performs real-time sleep staging and detection of large amplitude SOs. However, in this case, the tES is delivered following a 5-s delay from the negative peak of a SO. The purpose of the 5-s delay was to avoid interfering with ongoing SOs. In determining how long to wait before stimulation we considered a 1.5–2 s, “refractory period” that follows each SO (Schreiner et al., 2015; Farthouat et al., 2016), and the observation that SOs can occur in sequence. This did not completely preclude the possibility that tES could be delivered during a SO in some cases. The goal of the tES in our paradigm was not to enhance ongoing SOs, but rather to promote the subsequent occurrence of SOs. Compared to our previous study, where we used this closed-loop system with acoustic stimuli, we increased the minimum time between stimulation cycles to 30-s to allow for sufficient recovery from stimulation artifacts and to provide a longer window in between stimulation cycles for data analysis. As can be seen in Figure 2, a typical 30-s window containing a stimulation cycle will consist of about 8 s of stimulation artifact (4-s stimulation + 4-s recovery of EEG amplifiers) and 22 s of artifact-free EEG data before the next stimulation cycle can be triggered.

### Finite Element Modeling (FEM) of Induced Brain Currents

In order to confirm the presumed current path of the tES, we carried out a finite element modeling (FEM) procedure generally following that of Datta et al. (2009). Structural, T1-weighted, 3D volume MRIs were obtained from the Dayton Childrens’ Hospital using a 3.0 Tesla GE magnet with a standard clinical SPGR sequence (Davatzikos, 1996) in one participant. Four independent processes were merged to generate the FEM: segmentation, electrode generation and co-registration, tessellation, and calculation of the current distribution. The tissue segmentation was performed with the SPM12 Toolbox^2^ for MATLAB (Mathworks, Natick, MA, USA). The tissue segmentation is manually corrected by importing the T1 images and editing the tissue compartment masks in a graphical editor (ScanIP 6.0, SimpleWare, Herndon, VA, USA). The correction of the segmentation consisted of creating continuous and adjacent but non-overlapping boundaries for each of the seven tissue types; skin, bone, cerebrospinal fluid (CSF), gray matter (GM), white matter (WM), and air. Electrodes were created in a computer aided design program (Solidworks, Boston, MA, USA). The electrodes were individual disks, each 1.6 cm in diameter and 1 cm tall. Eight electrodes were placed on the surface of the scalp in the FEM model; two over the left mastoid, two over the right mastoid, two over the left frontal lobe around F3, and two over the right frontal lobe, around F4. The surfaces with co-registered electrodes were imported into ScanIP for creation of a volumetric mesh of tessellated tetrahedrons. The entire model and each tissue compartment within it was tessellated and the tetrahedral side length was scaled to give the final tessellated mesh 19–21 million elements. The tessellated mesh was imported into COMSOL (COMSOL Multiphysics 5.2, Stockholm, Sweden) for calculation of the current distribution in a FEM. In a detailed FEM, each tissue compartment is assigned a conductivity value in Siemens/meter (S/m) as shown in Table 2 (Datta et al., 2012). Additionally, the conductive value of air was set to 3 × 10^−15^ S/m and the value of the conductive gel that makes the connection between the Ag/AgCl electrode and the scalp was set to 4.0 S/m. While this provides a reasonably detailed model of the current distribution, it is still simplified compared to a real human head and results are therefore used primarily in doing gross localization of likely current deposition in the brain.

**Table 2:** Conductivity values (S/m) for the tissue layers in the tessellated volume for estimation of transcranial electrical stimulation (tES) current distribution (Datta et al., 2012).

WM	GM	CSF	Bone	Skin	Electrodes
0.126	0.276	1.650	0.010	0.465	1.400

*WM, white matter; GM, gray matter; CSF, cerebrospinal fluid*.

### Statistical Analyses

A 2 (Session: Post-Nap Test, Delayed Test) × 2 (Condition: sham and SDR-tES) repeated measure (RM) ANOVA was used to test the memory change across sessions and conditions. The sleep data were visually scored in 30-s epochs according to the American Academy of Sleep Medicine criteria (Berry et al., 2012). For the SDR-tES condition, we scored the epochs during the stimulation according to the following rules: (a) an epoch was visually staged if more than 50% of the epoch was stimulation artifact-free; and (b) if artifacts accounted for more than 50% of the epoch, the epoch was labeled using the subsequent sleep stage. This conservative approach is based on the assumption that if the stimulation disrupted sleep, the epoch following the stimulation will be a lighter stage or wakefulness. A 2 (Condition: sham and SDR-tES) × 4 (Stages: NREM1, NREM2, NREM3, REM) RMANOVA was run on the proportion of time spent in each sleep stage. Differences in the other sleep parameters [total sleep time (TST), sleep onset latency (SOL), wake after sleep onset (WASO), sleep efficiency (SE)] were analyzed with Wilcoxon Matched Pairs test. For the RM analyses, the Greenhouse-Geisser correction was applied where appropriate. In these cases, the uncorrected degrees of freedom and the adjusted *F*-values and probability levels were reported. Epsilon (ɛ) was reported as a measure of sphericity and partial eta-squared effect size ($\eta_{\text{p}}^{2}$) were reported as a measure of effect size. Fisher LSD test was used for *post hoc* analysis when needed. Associations between physiological measures—upstate duration of SOs, SO amplitude, SO rate, and slow (9–12 Hz) and fast spindle energy (12–15 Hz)—and memory performance were explored using Spearman’s Rho correlation.

All the other analyses were performed using the BioSig toolbox^3^ and EEGlab toolboxes^4^ (Delorme and Makeig, 2004) for MATLAB (MathWorks, Natick, MA, USA). For all sessions, EEG data were re-referenced to the linked mastoids and high-pass filtered at 0.2 Hz. In analyses requiring labeling of SOs we use the criterion described by Menicucci et al. (2009) for automated labeling of SOs during NREM2 and NREM3 sleep. In order to account for differences in participants’ EEG signal magnitude, we set the threshold for SO negative peak detection dynamically in each participant to 3*σ (the standard deviation of EEG in channels F3, Fz, F4 across all epochs of wake and NREM1 sleep). Using this approach results in thresholds of 72 ± 12 and 69 ± 11 μV for SDR-tES and sham conditions respectively (*t*_16_, *p* = 0.57). Power spectral density (PSD) was computed across all intervals of sleep (wake times were excluded) in each condition to investigate the impact of SDR-tES on the spectral power in the SO and the slow and fast spindle frequency bands. Slow and fast spindle energy were measured as z-scores within each spindle appropriate frequency band to reduce the effect of across participant variability in signal to noise for the smaller amplitude signals. Differences for fast and slow spindle energy were measured as a Cohen’s *d*. Statistics on SO energy and spindle and up state characteristics were performed using a paired samples *t*-test and were corrected for false discovery rate using the approach described in Benjamini and Hochberg (2000) using α < 0.05.

## Results

### Memory Performance

Table 3 shows the memory performance as a function of condition and testing session. No differences were observed between the two conditions at the Baseline Test (i.e., before the nap; *Z* = 0.59, *p* = 0.55).

**Table 3:** Memory performance across tests in the two conditions (*N* = 17).

	Sham	SDR-tES
**Test Performance**		
Baseline Test (%)	85.00 ± 10.00	83.24 ± 11.31
Post Nap Test (%)	74.71 ± 14.08	76.18 ± 10.24
Delayed Test (%)	49.12 ± 10.64	56.47 ± 13.78
**Performance change**		
Post Nap Performance Change (%)	87.71 ± 11.82	92.72 ± 14.23
Delayed Performance Change (%)	57.96 ± 11.63	67.75 ± 13.24

*The test performance is computed as the percentage of correctly recalled facts at each testing session. Performance change is computed for each participant as the performance at each test relative to the baseline test, with the baseline test set at 100%*.

**Figure 3:**
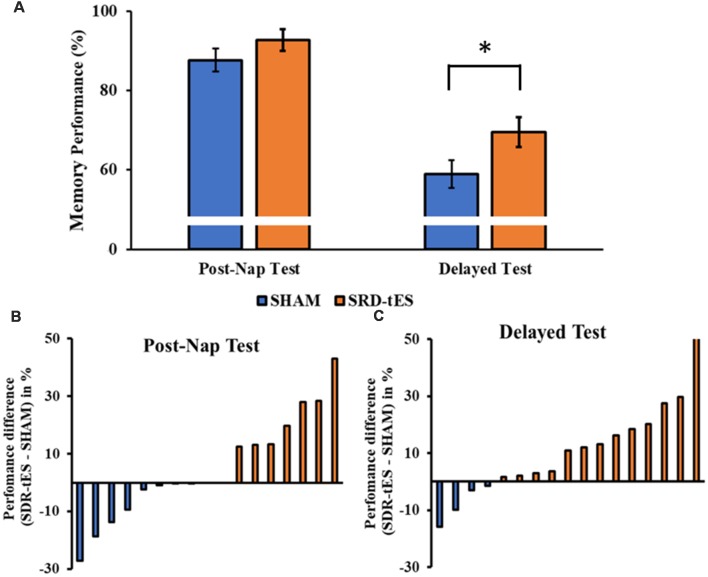
**(A)** The bars show the mean ± standard error of memory performance at each recall test relative to the baseline performance (with baseline set to 100%) in the sham and short duration repetitive (SDR)-transcranial electrical stimulation (tES) group. **(B,C)** The bars show individual’s performance difference between SDR-tES and sham conditions at the post-nap **(B)** and at the delayed test **(C)**. A positive value represents an advantage of SDR-tES whereas a negative value represents an advantage of sham. Most participants performed better in the SDR-tES condition, particularly at the delayed test. *p = 0.009.

The RM ANOVA highlighted a significant main effect of Session (*F*_(1,16)_ = 99.55, *p* < 0.001, $\eta_{\text{p}}^{2}$ = 0.86), with a decrease in memory performance in the Delayed Test compared to the Post-Nap Test, and a significant main effect of Condition (*F*_(1,16)_ = 7.55, *p* = 0.014, $\eta_{\text{p}}^{2}$ = 0.321), indicating greater memory retention for the SDR-tES condition compared to sham across the two tests. The lack of an interaction (*F*_(1,16)_ = 0.80, *p* = 0.384, $\eta_{\text{p}}^{2}$ = 0.043) was mainly due to the greater benefit of SDR-tES in both testing sessions (Figure 3A). We also decided to compute the individual performance differences between the SDR-tES and sham conditions. At the descriptive level, seven participants benefitted from the SDR-tES, and six from the sham at the Post-Nap Test (Figure 3B), whereas at the Delayed Test there was an evident benefit of the SDR-tES for 14 out of 17 participants (Figure 3C). We statistically confirmed these impressions: the difference between SDR-tES and sham was significantly larger than zero at the Delayed Test (*t*_16_ = 2.95, *p* = 0.009), but not at the Post-Nap Test (*t*_16_ = 1.14, *p* = 0.273).

### Sleep Architecture

Sleep summary is reported in Table 4. No significant differences were observed in TST (*Z*_(17)_ = 0.65, *p* = 0.518), SOL (*Z*_(17)_ = 0.52, *p* = 0.600), SE (*Z*_(17)_ = 0.78, *p* = 0.435) and WASO (*Z*_(17)_ = 1.76, *p* = 0.078).

**Table 4:** Sleep parameters in the two conditions.

	Sham	SDR-tES	Cohen’s *d*
TST (min)	77.50 ± 12.70	80.82 ± 13.61	−0.216
SOL (min)	3.97 ± 4.31	4.82 ± 6.46	−0.200
WASO (min)	11.79 ± 10.65	5.97 ± 5.16	0.537
SE (%)	82.80 ± 13.35	88.01 ± 9.40	−0.291
NREM1 (%)	20.27 ± 10.17	9.10 ± 6.35	1.069
NREM2 (%)	56.40 ± 15.78	48.41 ± 18.75	0.446
NREM3 (%)	19.30 ± 13.88	37.94 ± 18.37	−0.996
REM (%)	4.08 ± 6.89	4.82 ± 6.46	−0.057

*TST, total sleep time; SOL, sleep onset latency; WASO, wake after sleep onset; SE, sleep efficiency; REM, rapid-eye movement sleep*.

**Figure 4:**
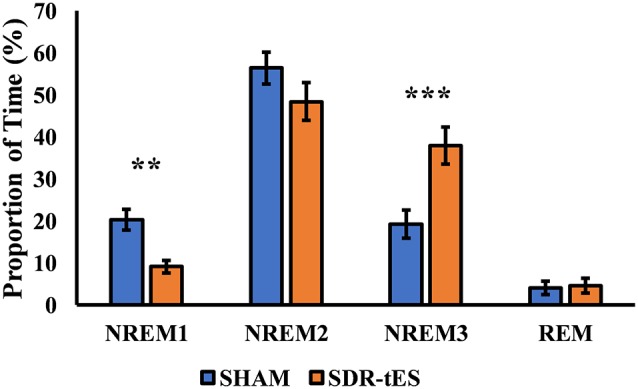
Proportion of time spent in each sleep stage in the two conditions. In the SDR-tES condition, there is an increased proportion of time in non-rapid eye movement 3 (NREM3) and a reduction of NREM1 sleep compared to the sham condition. Error bars represent the standard error of the mean. **p < 0.01; ***p < 0.001.

Although the main effect of Condition was not significant (*F*_(3,48)_ = 1.14, *ɛ* = 1.00, *p* = 0.301, $\eta_{\text{p}}^{2}$ = 0.066), the RM ANOVA showed a significant main effect of Stage (*F*_(3,48)_ = 46.48, *ɛ* = 0.52, *p* < 0.001, $\eta_{\text{p}}^{2}$ = 0.744) and a significant interaction between Stage and Condition (*F*_(3,48)_ = 10.77, *ɛ* = 0.59, *p* < 0.001, $\eta_{\text{p}}^{2}$ = 0.402). Fisher LSD *post hoc* tests showed a greater proportion of time spent in NREM3 and a lower proportion of time spent in NREM1 in the SDR-tES condition compared to the sham condition (*p* = 0.009 and *p* < 0.001, respectively, Figure 4). We also observed a reduced proportion of time spent in NREM2 in the SDR-tES, although only marginally significant (*p* = 0.056). Using a more conservative *post hoc* approach like Bonferroni (Lee and Lee, 2018), we still observe the increased proportion of time spent in NREM3 in the SDR-tES compared to the sham (*p* < 0.001), although the differences in NREM1 and NREM2 do not retain statistical significance using this test (*p* = 0.241 and *p* = 0.999, respectively).

### Neurophysiological Impact of SDR-tES

**Figure 5:**
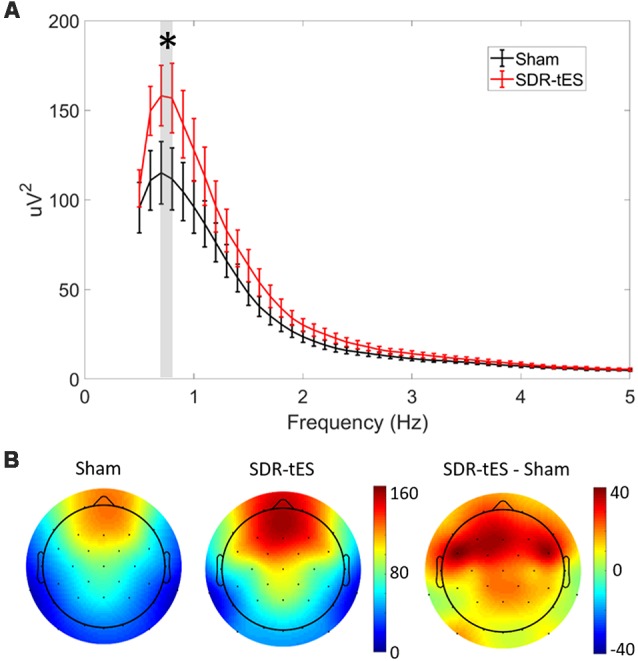
SDR-tES enhances SO spectral power. **(A)** Mean power spectral density (PSD) over the 0.5–5 Hz range at electrode Fz in sham and SDR-tES conditions. SDR-tES drives increases in the PSD around the stimulation frequency of 0.75 Hz (overlaying gray bar). Error bars indicate standard error of the means. *p < 0.05 (two-tailed, paired samples *t*-test). **(B)** The topoplots show the mean PSD at each electrode over the 0.7–0.8 Hz frequency range. The largest increases in PSD are over frontal electroencephalography (EEG) channels.

Figure 5A shows the mean PSD in channel Fz and Figure 5B shows the topology of the PSD around the stimulation center frequency (0.75 Hz) over the whole head for each treatment condition taken across both NREM2 and NREM3 sleep during the nap. SDR-tES results in an increase in the PSD at Fz near the peak stimulation frequency of 0.75 Hz (*t*_16_, *p* = 0.015). We did not observe any differences in PSD in either the slow (9–12 Hz) or fast (12–15 Hz) spindle bands. The topoplots show that the increases in PSD in the SO frequency band are largest near the anodes and the surrounding frontal electrodes. Differences across channels (*n* = 32) were not statistically significant after adjusting for false discovery rate. The larger PSD in the SO band supports the increases observed in NREM3 sleep. We next investigated whether SDR-tES impacts specific biomarkers in sleep associated with sleep-based memory consolidation.

**Figure 6:**
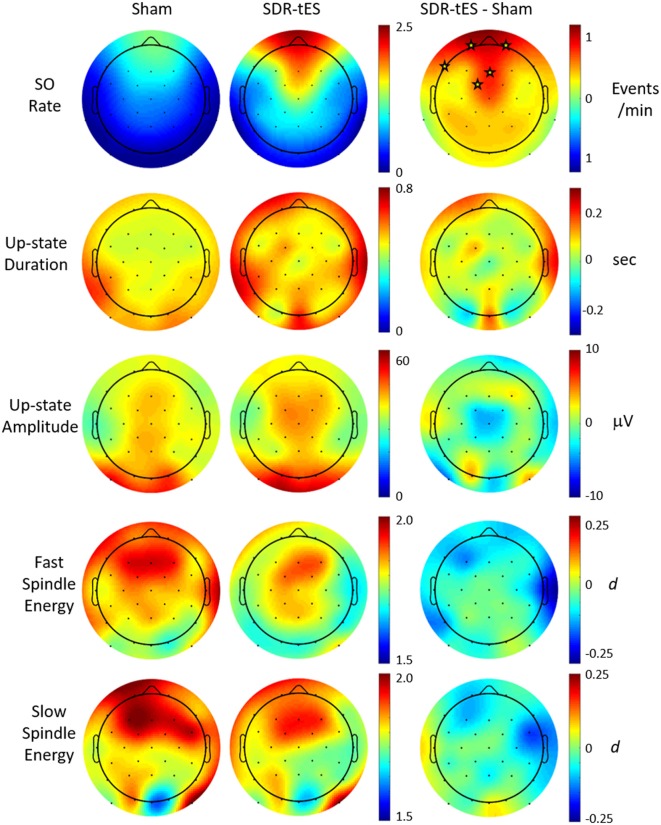
Effect of SDR-tES on down-to-up state transition-locked oscillatory measures related to memory consolidation during sleep. Each row shows the spatial distribution within each treatment group as well as the between-group difference for the given measure. SDR-tES results in significant increases in SO rate. Units for each measure are indicated to the right of each row. Fast and slow spindle energy were measured as z-scores within each spindle appropriate frequency band. Differences for fast and slow spindle energy are given as a Cohen’s *d*. Electrode locations marked with a star within each difference map indicate locations of statistical significance after adjusting for false discovery rate. The *p*-values for significant electrode locations are listed in Table 5.

Previous work has suggested that biomarkers for sleep-dependent memory consolidation may be strongest when nested in SO up states (Staresina et al., 2015). In order to look at differences in large amplitude SOs, we use previously described criterion for automatic labeling of SO events at each channel (Menicucci et al., 2009). Considering this set of SOs, we investigated the characteristics of the up states themselves as well as those of phase-locked sigma activity in the slow (9–12 Hz) and fast (12–15 Hz) spindle frequency bands. Figure 6 shows the topographical distribution of differences in the means of five different measures: SO rate, up state duration, up state amplitude, and fast and slow spindle energy during the up state.

Figure 6 shows that SDR-tES significantly increases the SO rate at frontal EEG channels where SOs are normally strongest. The spatial distribution in SO rate increases also indicates that these differences were smaller immediately adjacent to the stimulating anodes. The location, number and size of stimulating anodes in our system results in larger stimulation artifacts and longer recovery times relative to more distal electrode locations and this reduces the number of observed SOs at these sites. However, it is also possible that the effects of stimulation are not strongest immediately surrounding the stimulation sites and thus the observed differences in SO rates may represent real differences in stimulation efficacy. More work is needed to identify the source of this discrepancy. No significant differences were observed across any of the additional biomarkers investigated. Despite the lack of increase in mean spindle energy, the increase in overall SO rates would lead to a much larger increase in total phase-locked spindle energy due to the increase in the number of SOs. This should be taken into account when drawing any conclusions about spindle-related memory improvement.

**Figure 7:**
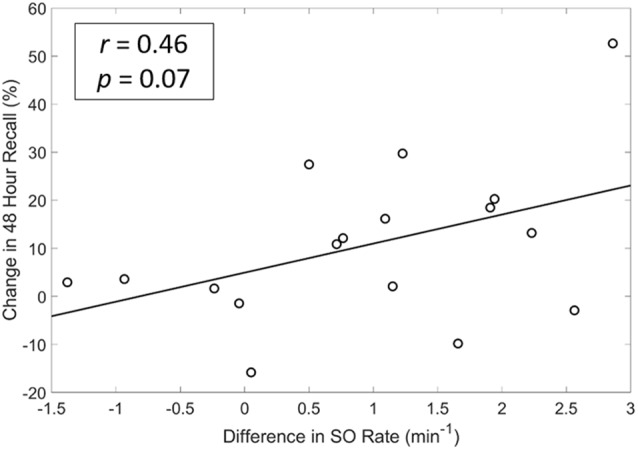
A modest and near significant positive correlation exists between differences in SO rate and memory recall at 48 h. *r* = Spearman’s Rho coefficient.

We also explored whether any of the neurophysiological measures described in Figure 6 (SO rate, upstate duration and amplitude of SOs, slow and fast spindle energy) were correlated with changes in memory performance. Specifically, we investigated the potential associations between changes in each participant’s test scores (SDR-tES—sham) and changes in each neural measure. For all measures except fast spindle energy, we averaged each measure across the Fp1, Fp2, and Fz electrodes where our observed changes in SOs were strongest. Given that fast spindles dominate parietal regions (Werth et al., 1997; Mölle et al., 2002, 2011), we used the average of P3, Pz, and P4 for this measure. Changes in SO rate were modestly correlated with change in memory recall 48 h after the initial learning (*r* = 0.46 *p* = 0.07, Spearman correlation, see Figure 7). Up-state duration (*p* = 0.66), up-state amplitude (*p* = 0.85), and fast and slow spindle energy measures (*p* = 0.89, *p* = 0.37, respectively) did not show any association with memory performance change. Similarly, there were no significant correlations between any measure and post-nap test performance where differences were smaller.

Given the possible connection between increases in the SO rate and memory performance, we sought to determine whether differences in the timing of stimulation relative to ongoing neural activity would have a predictable effect on the resulting increase in SOs. In order to investigate this, we looked retrospectively at the EEG activity immediately preceding each stimulation. Specifically, we investigated the correlation between the spectral power or cross-channel coherence in the 1 s prior to stimulation events (*n* = 417) and the number of SOs produced in the first 20 s after recovery from stimulation artifact in the subset of EEG channels which show statistically significant increases in SO rate (Fp1, Fp2, F7, Fz, FC1). Spectral power was measured in each channel and averaged across the set. Cross-channel coherence was assessed as the mean of the entire set of pairwise coherence values in the set. The 1-s window was chosen as the minimum amount of time that would enable assessment of lower frequencies (e.g., 1-Hz) while 20 s after the end of the stimulation artifact is almost the minimum duration before a subsequent stimulation cycle. Table 6 shows that several spectral bands including delta, beta, and gamma frequencies were significantly and negatively correlated with the resulting number of SOs immediately following stimulation in NREM3. Correlations between these measures and the resulting number of SOs in NREM2 are substantially weaker. Both spectral power and mean coherence show this negative correlation with stimulation efficacy and suggests that stimulating during more quiescent (low power) and less synchronized periods of brain activity lead to larger increases in the SO rate and conversely that stimulation during active brain processes and perhaps particularly during an ongoing SO, may reduce the number of resulting SOs.

**Table 5:** *P*-values for statistically significant channels with significantly greater slow oscillation (SO) rates in the short duration repetitive (SDR)-tES condition compared to sham (paired *t*-tests, adjusted for false discovery rate), as shown in Figure 6.

Channel	*p*-value
Fp1	0.002
Fp2	0.003
F7	0.013
Fz	0.015
FC1	0.007

**Table 6:** Spectral power and average cross-channel coherence in low and high-frequency bands during non-rapid eye movement 3 (NREM3) immediately preceding stimulation is negatively correlated with the response to stimulation.

	NREM3		NREM2	
EEG Measure	*r*	*p*-value	*r*	*p*-value
Delta power	−0.44	<0.001	0.10	0.03
Delta coherence	−0.38	<0.001	0.03	0.61
Theta power	0.10	0.08	0.03	0.50
Theta coherence	−0.03	0.41	0.16	0.004
Alpha power	−0.09	0.07	−0.11	0.01
Alpha coherence	−0.05	0.28	0.09	0.11
Sigma power	−0.14	0.004	−0.08	0.08
Sigma coherence	−0.18	0.06	0.001	0.98
Beta power	−0.17	<0.01	−0.02	0.61
Beta coherence	−0.33	<0.001	0.07	0.25
Gamma power	−0.19	<0.01	−0.05	0.25
Gamma coherence	−0.38	<0.001	0.16	0.004
Total power	−0.43	<0.001	0.08	0.08
Total coherence	−0.40	<0.001	0.18	0.002

### Finite Element Model Analysis

**Figure 8:**
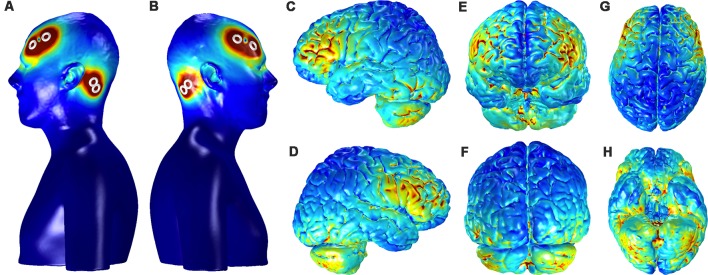
Current distribution on the scalp and the gray matter (GM) for the bilateral, fronto-mastoid electrode montage. Panels **(A,B)** show the left and right profiles, respectively, of scalp and skin showing the position of electrodes and the distribution of currents on the scalp (scale: 0–0.4 A/m^2^). Distribution currents on the surface of GM shown in the left and right hemispheres, **(C,D)** from anterior and posterior views, **(E,F)** and from dorsal and ventral views, **(G,H)**. The scale for the images depicting GM, **(C–H)** is 0–0.05 A/m^2^.

Figure 8 shows the current distribution across the cortical surface and ventral brain structures in a single participant used for FEM. The regions of strongest current deposition appear to be on bilateral frontal cortices approximately under the anodes with weaker activation more posterior towards the cathodes. Strong bilateral frontal activation is consistent with the increases in slow oscillatory power observed over frontal electrodes and provides further evidence that the stimulation may be directly affecting neurophysiology to directly modulate the cortical SO. The model also suggests that some of the current is distributed to the ventral portions of the temporal lobe and the cerebellum. Possible effects of any current in these structures are unknown.

## Discussion

In the current study, we tested a novel approach for delivering tES during sleep we termed SDR-tES, wherein we delivered short bursts of slow oscillatory tDCS following a SO event. Similar to previous studies using tES during sleep, we observed that this approach improved consolidation of declarative memory compared to a sham condition. SDR-tES also increased the relative amount of NREM3 sleep at the cost of NREM1 sleep. Neurophysiologically, SDR-tES enhanced the rate of SOs, but not sleep spindle activity during SO upstates. We also found that the number of SOs immediately following SDR-tES was negatively correlated with several measures of spectral power and coherence suggesting that stimulation may be more effective when applied during more quiescent (low power) and less synchronized periods of brain activity. The current results demonstrate that SDR-tES is a feasible approach to improve memory-related sleep physiology and declarative memory consolidation and extend the use of tES during sleep to enhance memory and learning.

The current protocol extends well-known literature on electrical stimulation during sleep. Marshall et al. (2006) were the first to show that frontal slow oscillatory stimulation enhanced SO and spindle activity compared to sham stimulation. Moreover, they observed that the stimulation enhanced participants’ performance on a paired-association task. Similar findings were reported using comparable protocols and different tasks (e.g., word list learning task, visual paired associated task; Marshall et al., 2004; Antonenko et al., 2013; Göder et al., 2013; Prehn-Kristensen et al., 2014; Ladenbauer et al., 2016). Additionally, the same approach has been successful in enhancing declarative memory for word-pairs in older adults (Eggert et al., 2013), with one study also showing increased SO activity after slow oscillatory tDCS during a daytime nap in elderly (Westerberg et al., 2015). Interestingly, despite different statistical results (i.e., different *p*-values), the effect size of stimulation benefit was similar in these two studies, indicating a superior performance after the slow oscillatory tDCS compared to sham (see Barham et al., 2016). The present results replicate and build on this body of work by applying short durations of repetitive tDCS delivered throughout NREM2/NREM3 resulting in decreased forgetting for recently learned facts. We also showed that tES stimulation markedly increases the rate of SOs over frontal EEG channels. Together with the current study, these results indicate that slow oscillatory tDCS can be effective at modulating the SO, and provides confirming evidence for the active role of SOs in declarative memory consolidation. At first glance, our results appear in direct contrast to a recently published study that demonstrated closed-loop tACS phase-locked to ongoing SO reduced the SO rate (Ketz et al., 2018). However, the negative correlation between delta power/coherence and the resulting number of SOs observed in our study, suggests that when stimulation coincides with ongoing slow wave activity it may, in fact, result in fewer SOs as was similarly demonstrated by Ketz et al. (2018). Our results, therefore, suggest that if the goal is to increase SOs, a better closed-loop approach may be the targeting of asynchronous and quiet periods of neural activity during NREM3 sleep.

Examination of the electrophysiological signatures of the stimulation, compared with sham, identifies several features that deserve discussion. Here we showed a marked increase in the occurrence of SOs, but no effect of stimulation on SO amplitude. However, we did not observe any significant increase in the power of these oscillations (SO, slow and fast spindles) after stimulation. The lack of effect may be due to several reasons, including the timing of our stimulation (5 s after an endogenous SO) and/or a ceiling effect caused by the task itself, which may have induced a strong increase in spindle response even in the sham condition. It is worth noting that although the mean spindle energy per SO was not statistically enhanced, the higher SO rate in the SDR-tES condition very likely results in a greater number of SO-coupled spindle activations, which is considered a key mechanism for memory consolidation during sleep (Antony et al., 2018; Bergmann and Born, 2018; Helfrich et al., 2018). Thus, it may be that SDR-tES increases the number of opportunities (SOs) for memory consolidation rather than strongly increasing the efficacy of existing opportunities.

Compared to the original work by Marshall et al. (2006), our approach used shorter durations of stimulation more regularly triggered by the underlying sleep physiology. We began our study with an estimation of sample size based on a power analysis of her seminal study (*n* = 13). The effect sizes observed in our memory tasks are slightly smaller (cohen’s *d* = 0.74) suggesting that 22 participants would ultimately be required for a fully powered behavioral result (*β* = 0.08, *α* = 0.05). The high demand of our protocol and the requirement of multiple successful naps dictated by our within-subjects design, unfortunately, resulted in a high attrition rate among our relatively large cohort (*n* = 57) of enrolled participants. Results on the enhancement of NREM3 and SO rate are fully powered in the current cohort. Future use of the SDR-tES approach should investigate to what extent the parameters of duration and delay in the stimulation contribute to or change the observed effects and should also investigate the impact of closed-loop delivery using different brain activity triggers such as the low power and coherence conditions described here.

Another shortcoming of this and all prior work using tES is an inability to observe the direct response during stimulation. Elimination of the stimulation artifact would enable a more direct comparison of results across studies in terms of the immediate impact of the intervention. A potential benefit of our approach is a large reduction in the total dose of stimulation during sleep. Additionally, this stimulation protocol was successfully applied during a daytime nap, when our participants do not reliably spend a significant amount of time in NREM3 sleep. The fact that significant neurophysiological effects (including the increases in SO rate) are observed even with a limited period of stimulation suggests that the stimulation dose required for benefit may be much less than the 20–60 min maximum duration suggested for minimal risk (Fregni et al., 2015). Practically, this reduced amount of stimulation could encourage longer-term use of this approach. Moreover, the significantly increased time in NREM3 with stimulation may be of interest in older adult populations who have reduced deep sleep (Van Cauter et al., 2000; Yetton et al., 2018) in particular during daytime sleep (Sattari et al., 2019).

A novel element of the current study is the learning task used to probe declarative memory. Previous studies, not only in the tES and sleep literature, but in the overall sleep field, have used standardized laboratory tasks (e.g., word-pair association tasks, object location tasks). In this study, we designed a declarative task which required participants to learn a series of facts about destinations around the world. The task resembles a presentation style used in most standard educational paradigms and the format is amenable to almost any type of learning material. As such, the task is highly ecologically valid and suggests strongly that the use of SDR-tES may be a useful intervention for improving memory in more standard educational tasks. However, extensive research will be required to validate the longer-term consequences of this type of intervention on both neurophysiology and behavior prior to use in these settings.

In conclusion, we have shown that SDR-tES is a feasible approach to improve memory-related sleep physiology and declarative memory consolidation of facts. This improvement can be achieved after a single daytime nap period and with a reduced amount of stimulation compared to standard open-loop stimulation approaches.

## Ethics Statement

The study was approved by the New England Independent Review Board and all participants gave written informed consent in accordance with the Declaration of Helsinki.

## Author Contributions

SS, PC, MA-S, MW, NC, and SM designed the experiment. RS, DA, NC, AP, and LH collected the data. RE, MA-S, and SS developed the algorithms. SS and NC performed the statistical analyses. NC, RS, SM, and SS wrote the manuscript. All authors contributed to the manuscript revision and approved the submitted version.

## Conflict of Interest Statement

Teledyne Scientific & Imaging has filed a patent application (no. 62/403318) based on parts of the described research. RS, PC, AP, RE, MA-S, and SS were employed by Teledyne Scientific & Imaging. DA and MW were employed by Rio Grande Neurosciences. The remaining authors declare that the research was conducted in the absence of any commercial or financial relationships that could be construed as a potential conflict of interest.

## Footnotes

^1^http://openvibe.inria.fr

^2^http://www.fil.ion.ucl.ac.uk/spm/software/spm12/

^3^http://biosig.sourceforge.net/index.html

^4^https://sccn.ucsd.edu/eeglab/index.php
